# Diagnostic performance of CT for extrarenal fat invasion in renal cell carcinoma: a meta-analysis and systematic review

**DOI:** 10.1186/s13244-024-01889-0

**Published:** 2025-01-15

**Authors:** Junchao Ma, Enyu Yuan, Shijian Feng, Jin Yao, Chunlei He, Yuntian Chen, Bin Song

**Affiliations:** 1 Department of Radiology, West China Hospital, Sichuan University, Chengdu, China; 2 Department of Urology and Institute of Urology (Laboratory of Reconstructive Urology), State Key Laboratory of Biotherapy and Cancer Center, West China Hospital, College of Life Sciences, Sichuan University, Chengdu, China; 3 Department of Radiology, Sanya People’s Hospital, Sanya, China

**Keywords:** Renal cell carcinoma, Perinephric fat invasion, Renal sinus fat invasion, Meta-analysis, Computed tomography

## Abstract

**Objectives:**

Renal cell carcinoma (RCC) with extrarenal fat (perinephric or renal sinus fat) invasion is the main evidence for the T3a stage. Currently, computed tomography (CT) is still the primary modality for staging RCC. This study aims to determine the diagnostic performance of CT in RCC patients with extrarenal fat invasion.

**Methods:**

The PubMed, Web of Science, Cochrane Library, and EMBASE databases were systematically searched up to October 11, 2023. Study quality was assessed by the QUADAS-2 tool. Standard methods recommended for meta-analyses of diagnostic evaluation were used. Heterogeneity was analyzed through meta-regression analysis.

**Results:**

Fifteen studies were included in this meta-analysis. Among them, six studies focused on perinephric fat invasion (PFI) only, four on renal sinus fat invasion (RSFI) only, and five on both. Pooled weighted estimates of sensitivity, specificity, area of SROC curve, PLR, and negative likelihood ratio (NLR) of CT for PFI were 0.69 (95% CI: 0.55–0.79), 0.82 (95% CI: 0.69–0.90), 0.81 (95% CI: 0.77–0.84), 3.85 (95% CI: 2.22–6.67), and 0.38 (95% CI: 0.27–0.55). Pooled weighted estimates of sensitivity, specificity, area of SROC curve, PLR, and NLR of CT for RSFI were 0.81 (95% CI: 0.76–0.85), 0.79 (95% CI: 0.66–0.88), 0.82 (95% CI: 0.78–0.85), 3.91 (95% CI: 2.26–6.77), and 0.24 (95% CI: 0.18–0.31).

**Conclusion:**

CT has the ability to detect the PFI and RSFI in patients with RCC. However, the diagnostic performance of CT has suffered from the limitation of slightly lower accuracy, resulting from the low positive sample in the current studies. Additionally, the current PLR is low.

**Critical relevance statement:**

This study provides radiologists and urologists with a systematic and comprehensive summary of CT and CT-related morphological features in assessing extrarenal fat invasion in patients with RCC.

**Key Points:**

- CT can detect extrarenal fat invasion in patients with RCC, but the diagnostic performance is inconsistent.
- The diagnostic performance of CT is acceptable, but primarily affected by the low positive rate of included patients.
- Further large-scale trials are necessary to determine the true diagnostic capabilities of CT for extrarenal fat invasion.

**Graphical Abstract:**

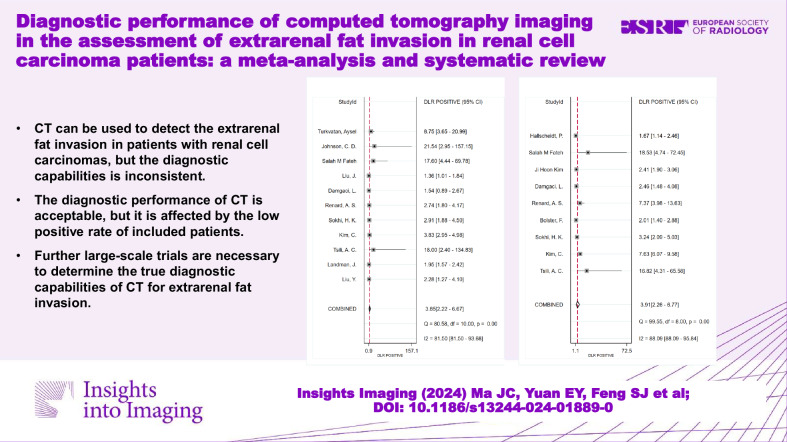

## Introduction

Based on the 8th edition of the American Joint Committee on Cancer Tumor Node Metastasis (TNM) staging guidelines, renal cell carcinoma (RCC) involving the renal vein, perinephric fat, renal sinus fat, or the pelvicalyceal system is classified as T3a [1, 2]. The T stage is crucial for risk stratification in RCC, particularly T3a, which significantly influences clinical decision-making and prognostic evaluation [3, 4]. As the main evidence for the T3a, extrarenal fat (perinephric or renal sinus fat) invasion represents a high-risk subgroup associated with a poorer prognosis, necessitating more aggressive therapeutic strategies [5, 6]. According to the European Association of Urology Guideline Group for RCC, the standard treatment for locally advanced (T3a–T4) RCC is radical nephrectomy, performed via laparotomy or laparoscopy [7]. For localized stages (T1a–T2b), nephron-sparing surgery is preferred, reducing the risk of cardiovascular and metabolic disorders by preserving renal function [8–10]. The infiltration of the renal capsule or the existence of neoplastic cells having direct contact with the perinephric fat was characterized as a perinephric fat invasion (PFI) [11]. Unlike perinephric fat, there is a lack of clear fibrous capsules between the inner margin of the kidney and the renal sinus [12]. The renal sinus fat is the central fat compartment separating the collecting system and the renal parenchyma, which contains abundant lymphatics, vascular structures, and fibrous tissue [13]. Due to its anatomical implications, renal sinus fat invasion (RSFI) typically predicts a worse prognosis, with any form of extrarenal invasion correlating with a 5-year cancer-specific survival rate between 50.6% and 68.1% [14–22]. Thus, precise pre-treatment determination of extrarenal fat invasion is vital for providing prognostic insights and optimizing patient management strategies.

Computed tomography (CT) remains the primary modality for clinically staging RCC [2, 23–25]. Previous studies have identified various imaging features that help radiologists assess T3a invasions, with PFI and RSFI being the most frequently studied. However, different prediction performances and even contradictory results were found in various studies. Notably, perinephric soft-tissue stranding, characterized by linear soft-tissue attenuation in perinephric fat, demonstrated high sensitivity (91.6–93.3%) but low specificity (36.1–52.1%) in some studies [26–28]. Another study, however, reported lower sensitivity (71.4%) and higher specificity (80.6%) for this feature [13]. Thus, deriving a reliable and objective conclusion is essential to prevent both overtreatment and undertreatment, particularly concerning PFI and RSFI.

This meta-analysis aims to evaluate the diagnostic accuracy in detecting PFI and RSFI by radiologists using CT in patients with RCC, and to assess the diagnostic value of related morphological features.

## Materials and methods

This meta-analysis adhered to the preferred reporting items for systematic reviews and meta-analyses of diagnostic test accuracy studies guidelines [29, 30]. The study protocol was registered and published on the International Platform of Registered Systematic Review and Meta-analysis Protocols (INPLASY) under the registration number INPLASY202430017. Institutional Research Ethics Board approval was not required for this study.

### Literature search

A comprehensive systematic search was conducted across PubMed, Web of Science, EMBASE, and the Cochrane Library databases on September 10, 2023, utilizing medical subject headings and entry terms of related keywords without imposing any date or language restrictions. To capture all pertinent literature, the keyword “T stage” was included, acknowledging that some studies on the accuracy of CT in T staging might encompass data on T3a and its subcategories. Additional sources were identified by reviewing relevant reviews, original studies, and their reference lists. The search was last updated on January 14, 2024. A detailed search strategy is documented in Supplementary Table 1.

### Study selection

Titles, abstracts, and full texts of all retrieved publications were independently screened by two reviewers. Any discrepancies were resolved through consensus. The inclusion criteria were: (a) studies where CT was employed to evaluate PFI and RSFI in RCC patients, and the subtype of RCC was not restricted; (b) assessments of the aforementioned invasions by CT were restricted to qualitative sign analysis and semi-quantitative data measurement by radiologists; (c) postoperative histopathological analysis served as the reference standard for detecting extrarenal invasions.

Non-original studies such as academic reviews, systematic reviews, and case reports were excluded. Additionally, the exclusion criteria included: (a) studies involving minors, predominantly concerning Wilms’ tumor; (b) studies on renal capsule invasion, which is distinct from renal capsule infiltration; (c) since this review aims to evaluate the diagnostic accuracy of radiologists, studies employing texture analysis, radiomics, and deep learning were excluded, except in cases where human interpretation served as a comparator; and (d) insufficient data to construct a 2 × 2 contingency table.

### Data extraction

Data were independently extracted from the included studies by two reviewers, with any discrepancies resolved through consensus and discussion involving a third reviewer. The extracted data for each study included the first author, year of publication, tumor staging criteria and its version (TNM system and Robson classification), CT imaging features or criteria for positive cases, subsets of features, number of patients, number of lesions, number of positive cases, proportion of positive cases, number of true positives, false positives, false negatives, and true negatives, as well as sensitivity, specificity, number of readers, consensus read, and the use of excretory phase images. For studies involving multiple readers, the average of the diagnostic performance was calculated and analyzed. In studies featuring different CT imaging features, priority was given to signs identified through multivariate regression analysis or those most aligned with clinical practice for comprehensive analysis.

### Quality assessment

Risk of bias and applicability concerns were evaluated using the quality assessment of diagnostic accuracy studies-2 (QUADAS-2) tool by two reviewers. This tool assesses the risk of bias across four domains: study selection, index test, reference standard, and flow and timing. Applicability concerns are examined in three domains: patient selection, index test, and reference standard [31]. Any disagreements were resolved through consensus and discussion with the third reviewer.

### Statistical analysis

Pooled summary estimates for sensitivity, specificity, diagnostic odds ratio (DOR), negative likelihood ratio (NLR), and positive likelihood ratio (PLR) were derived using a bivariate random effects model that included all studies and subsets of CT features and criteria. We assessed potential heterogeneity among the studies using Cochran’s Q-statistic and the *I*^2^ tests. The area under the curve (AUC), representing the summary curve of diagnostic performance, was obtained through the hierarchical summary receiver operating characteristics (HSROCs) model. Univariate meta-regression was conducted based on variables such as publication year, number of patients, number of readers, number of CT imaging features or criteria, consensus read, and excretory phase, which were anticipated as potential sources of heterogeneity. The presence of publication bias in the included studies was evaluated using Deek’s funnel plot. Statistical analyses were performed using STATA version MP 16.0 (stataMP), with a significance level set at *p* < 0.05. Quality assessment was performed using Review Manager 5.3.

## Results

### Literature search and study selection

**Fig. 1:**
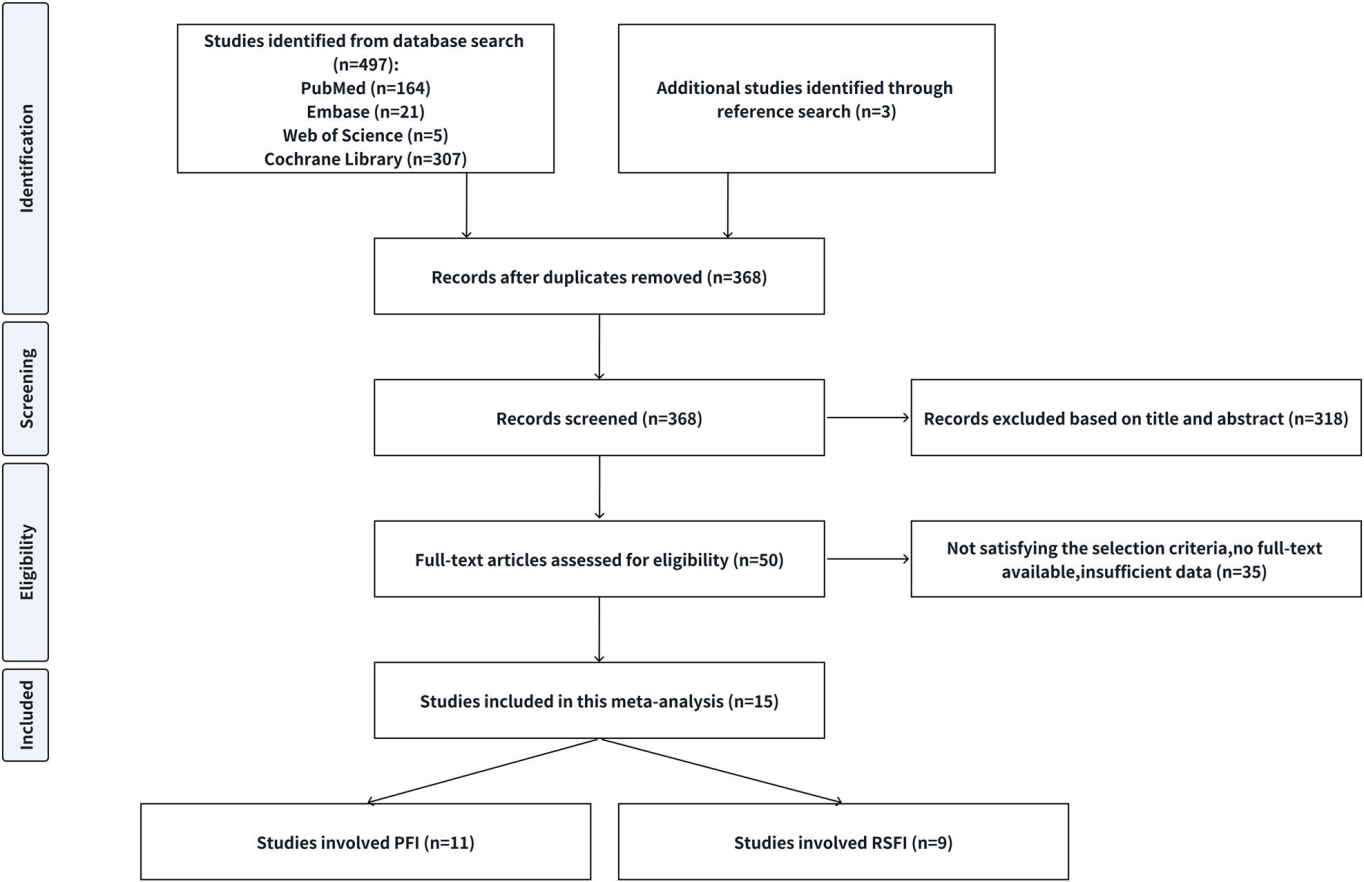
Flowchart of the study selection process for the meta-analysis

A systematic search initially identified 497 studies, of which 318 were excluded based on title and abstract review, and 35 were further excluded after full-text assessment. Fifteen studies met the inclusion criteria and were subsequently included in the meta-analysis. Among these, 6 studies focused on PFI only, 4 on RSFI only, and 5 on both. A flow diagram illustrating the study selection process is depicted in Fig. 1. Of the included studies, only three were prospective. The 11 studies addressing PFI encompassed 1535 patients and 1545 RCC lesions, while the 9 studies on RSFI involved 1617 patients and 1624 RCC lesions. In all studies, the assessment of extrarenal fat invasion was validated through histopathological analysis.

### Data extraction and quality assessment

**Fig. 2:**
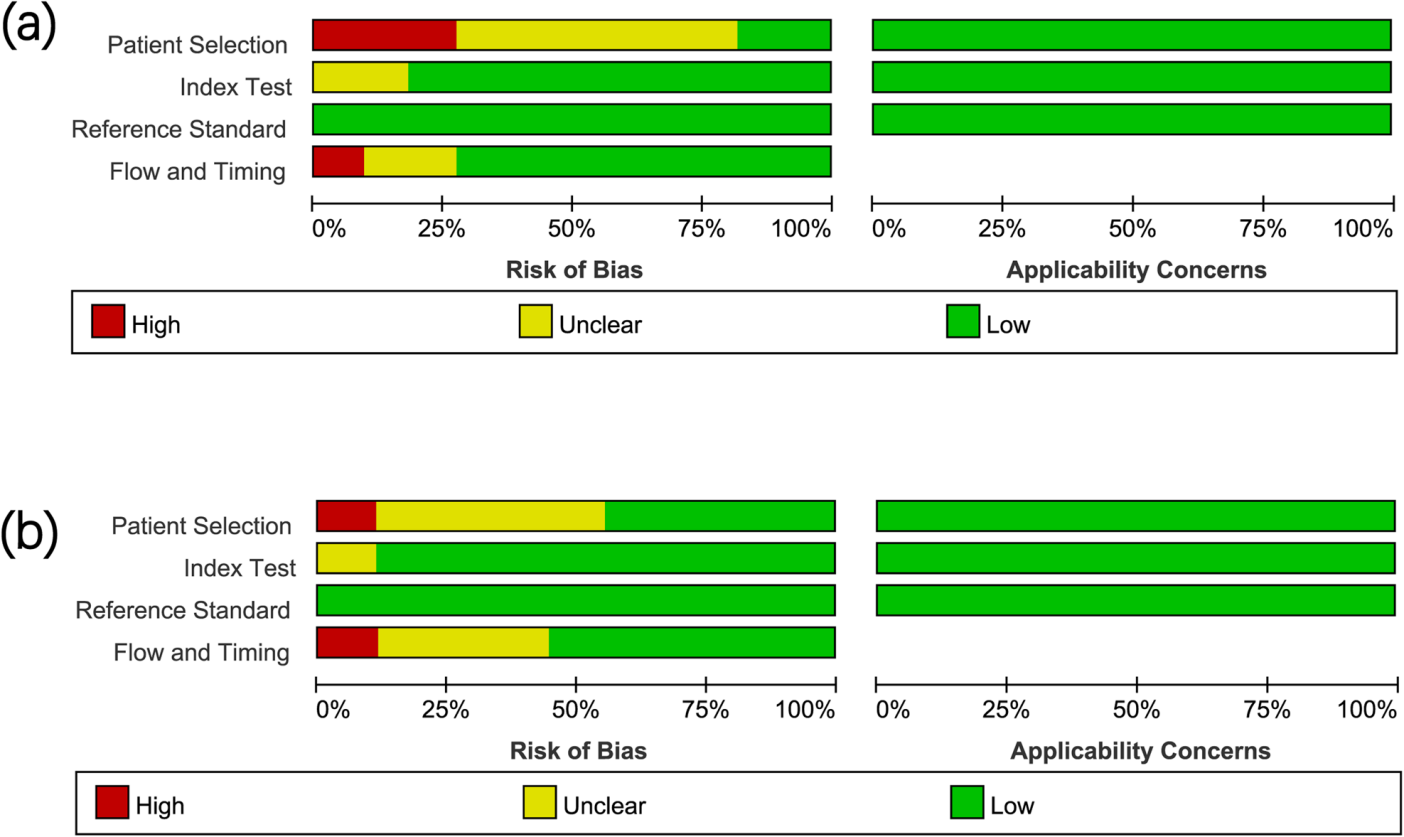
**a** Overall quality assessment of the included studies on PFI using the QUADAS-2 tool. **b** Overall quality assessment of the included studies on RSFI using the QUADAS-2 tool

The characteristics of the studies related to extrarenal fat invasion are detailed in Tables 1 and 2, and Supplementary Tables 3 and 4, respectively. Using the QUADAS-2 tool, the assessment of the risk of bias and applicability concerns for studies on PFI and RSFI is depicted in Fig. 2a, b, respectively. For PFI studies, three studies exhibited a high risk of bias in patient selection due to nonconsecutive enrollment, case-control design, and exclusion of specific cases (patients with bilateral RCC were excluded) [11, 28, 32]; six studies had an unclear risk because they either did not specify exclusion criteria or only detailed the timing of case inclusion without confirming consecutive enrollment [13, 27, 33–36]. The risk of bias in flow and timing was high in one study due to the analysis including only a subset of patients with pathological data on PFI [36]; two studies presented an unclear risk because the time interval between CT examination and surgery was not specified [13, 34]. For RSFI, nonconsecutive enrollment primarily contributed to a high risk of bias in patient selection in one study [28]. The unclear risk of bias in four studies was due to similar reasons as those mentioned for PFI [13, 35, 37, 38]. One study excluded patients with papillary RCC and chromophobe RCC; however, since clear cell carcinoma is the predominant RCC type, the potential bias introduced by these exclusions remains uncertain. The clarity of flow and timing assessments was compromised in three studies due to unspecified time intervals [12, 13, 39], and one prospective study showed a high risk of bias because it included only a partial cohort in the analysis—some patients were either clinically unfit for surgery or were not confirmed to have RCC by pathology [38].

**Table 1:** The characteristics of the included studies involved the PFI

First Author [Reference No.]	Year	T Staging Criteria	Features	Subsets of Features	No. of Patients	No. of PFI	TP	FP	FN	TN	Sensitivity	Specificity	No. of Patients > 100	Published in 2017 and later	No. of Readers > 1	No. of Features > 1	Consensus Read	Excretory Phase images
Liu et al [11]	2023	TNM 2009	PNSS+PNSN+ITM+PTV+PNFD	PNSD and Others	131	64	43	33	21	34	67.50%	50.00%	Yes	Yes	Yes	Yes	NM	NM
Damgaci et al [13]	2021	TNM 2017	PNSS+ITM+PTV	PNSD and Others	50	14	9	15	5	21	64.00%	58.00%	No	Yes	No	Yes	No	No
Liu et al [27]	2012	TNM 2009	PNSS+PNSN	PNSD	312	31	10	40	21	243	32.26%	85.87%	Yes	No	No	Yes	No	Yes
Tsili et al [28]	2013	TNM 2010	PNSN	PNSD	47	12	6	1	6	35	50.00%	97.20%	No	No	Yes	No	Yes	Yes
Landman et al [32]	2017	Not Mentioned	PNSS	PNSD	161	15	14	70	1	76	93.30%	52.10%	Yes	Yes	Yes	No	NM	Yes
Türkvatan et al [33]	2009	TNM 1997	PNSS+PSSN	PNSD	57	3	3	5	0	49	100.00%	91.00%	No	No	Yes	Yes	Yes	No
Kim et al [34]	2014	TNM 2009	-	-	408	20	17	86	3	302	82.50%	77.85%	Yes	No	Yes	Yes	Yes	Yes
Fateh et al [35]	2023	TNM 2017	PNSS+ITM	PNSD and Others	59	15	12	3	4	42	80.00%	95.45%	No	Yes	No	Yes	No	Yes
Johnson et al [36]	1987	Robson staging	PNSN	PNSD	97	24	11	1	13	46	46.00%	98.00%	No	No	No	Yes	No	Yes
Sokhi et al [40]	2015	TNM 2009	PNSN+ITM	PNSD and Others	117	16	12	26	4	75	75.50%	74.00%	Yes	No	Yes	Yes	No	No
Renard et al [41]	2019	TNM 2009	PNSS	PNSD	96	22	17	22	5	56	77.00%	72.00%	No	Yes	Yes	No	No	Yes

*PNSS* perinephric soft-tissue stranding, *PNSN* perinephric soft-tissue nodule, *PNSD* perinephric soft-tissue density, *ITM* irregular tumoral margin, *PTV* peritumoral vessels, *PNFD* perinephric fat density, *TP* true positive, *FP* false positive, *FN* false negative, *TN* true negative, *NM* not mentioned

**Table 2:** The characteristics of the included studies involved the RSFI

First Author [Reference No.]	Year	T Staging Criteria	Features	Subsets of Features	No. of Patients	No. of RSFI	TP	FP	FN	TN	Sensitivity	Specificity	No. of Patients > 100	Published in 2017 and later	No. of Readers > 1	No. of Features > 1	Consensus Read	Excretory Phase
Kim et al [12]	2014	TNM 2009	-	-	863	110	88	79	22	674	79.55%	89.45%	Yes	No	Yes	Yes	Yes	Yes
Damgaci et al [13]	2021	TNM 2017	Extension into RS+ Compression/invasion of PS	RSS	50	7	6	15	1	28	85.70%	65.10%	No	Yes	No	Yes	No	No
Tsili et al [28]	2013	TNM 2010	PSI	RSS	47	11	10	2	1	35	90.90%	94.60%	No	No	Yes	No	Yes	Yes
Fateh et al [35]	2023	TNM 2017	PSI+RSFI	RSS	59	17	15	2	2	40	88.23%	95.23%	No	Yes	No	Yes	No	Yes
Bolster et al [37]	2016	TNM 2009	Abut or bulge into the RS	RSS	53	19	18	17	1	19	96.85%	52.50%	No	No	Yes	Yes	No	No
Hallscheidt et al [38]	2006	TNM 2002	ITM	Others	76	4	4	28	0	24	87.50%	47.00%	No	No	Yes	No	No	No
Kim et al [39]	2021	TNM 2017	ITM	Others	276	81	62	62	19	133	77.15%	67.95%	Yes	Yes	Yes	NO	Yes	No
Sokhi et al [40]	2015	TNM 2009	SN+ITM within the SF	Others	117	47	37	17	10	53	79.50%	75.00%	Yes	No	Yes	Yes	No	No
Renard et al [41]	2019	TNM 2009	SS near the PSI	Others	96	15	13	10	2	75	86.00%	88.00%	No	Yes	Yes	No	No	Yes

*PSI* pelvicaliceal system invasion, *RSS* renal sinus structure, *RS* renal sinus, *PS* pelvicaliceal system, *RSFI* renal sinus fat invasion, *ITM* irregular tumoral margin, *SN* soft-tissue nodule, *SF* sinus fat, *SS* soft-tissue stranding, *TP* true positive, *FP* false positive, *FN* false negative, *TN* true negative, *NM* not mentioned

No studies on extrarenal fat invasion were found to have a high risk of bias regarding the index test; however, one study was deemed unclear because it did not specify whether the assessment was blinded [35]. The risk of bias in the reference standard, along with applicability concerns for patient selection, index test, and reference standard, were low across all included studies.

### CT assessment for PFI

**Fig. 3:**
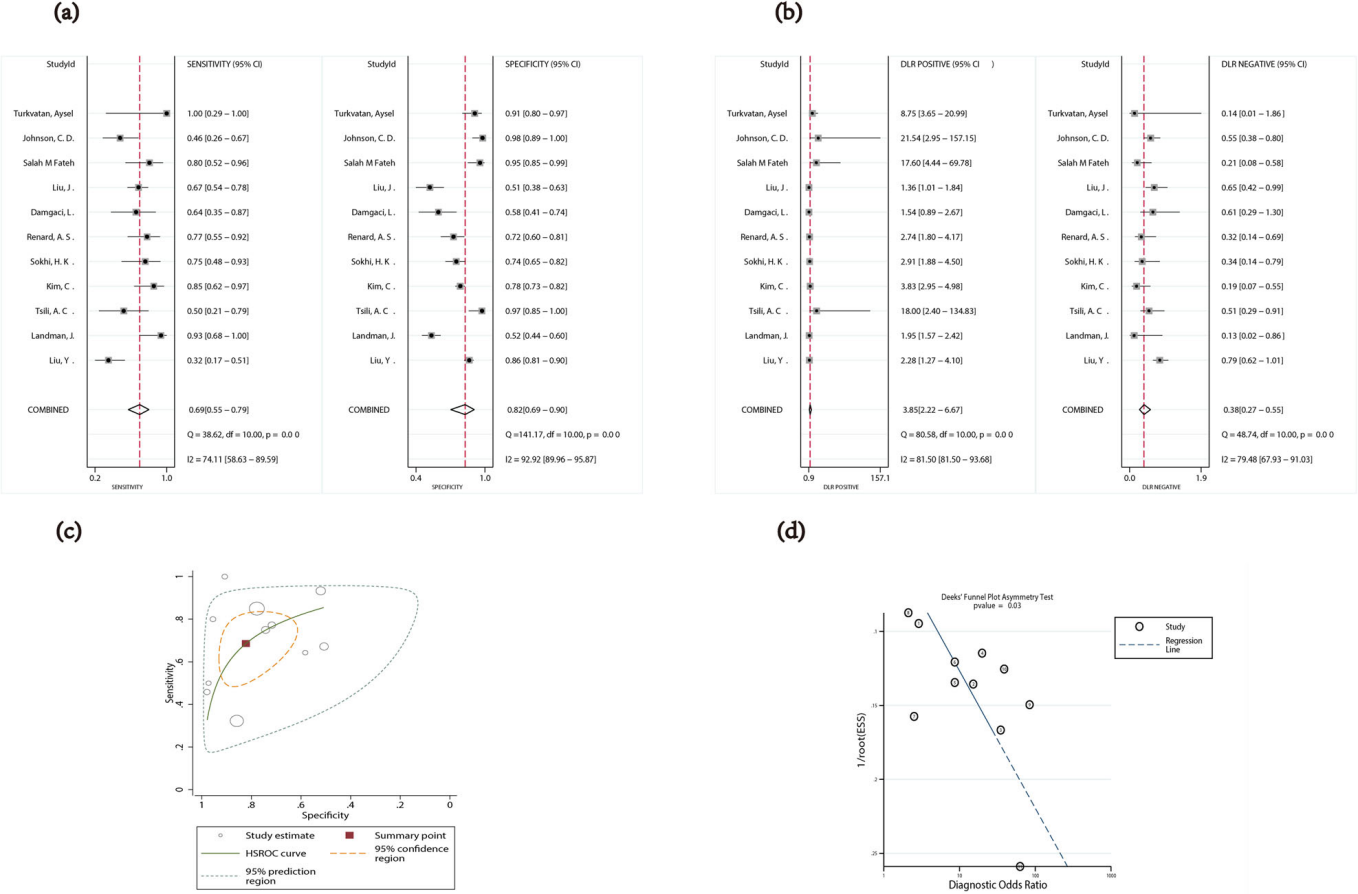
**a** Forest plot of sensitivity and specificity of CT assessment for PFI. **b** Forest plot for likelihood ratio after combination (LR+, LR−). **c** The HSROC curve of CT assessment for PFI (HSROC hierarchical summary receiver operating characteristic). **d** The Deek funnel plot of studies on PFI

The sensitivity (*p* < 0.05, *I*^2^ = 74.11%) and specificity (*p* < 0.05, *I*^2^ = 92.92%) of CT assessment for PFI showed significant heterogeneity, with a pooled sensitivity of 0.69 (95% CI: 0.55–0.79) and a pooled specificity of 0.82 (95% CI: 0.69–0.90) (Fig. 3a). The combined PLR, NLR, DOR, and diagnostic score were 3.85 (95% CI: 2.22–6.67), 0.38 (95% CI: 0.27–0.55), 10.06 (95% CI: 4.82–21.00), and 2.31 (95% CI: 1.57–3.04), respectively (Fig. 3b and Supplementary Fig. 1). The area under the summary receiver operating characteristic (SROC) curve was 0.81 (95% CI: 0.77–0.84) (Fig. 3c). The accuracy parameter (Lamda) was 2.20 (95% CI: 1.46–2.95).

Further analysis of heterogeneity was undertaken by univariate meta-regression, which suggested that the factors influencing the diagnostic performance included the publication year and number of patients. They showed the same significant effect on specificity. The five studies published with 100 or more cases had a 70% pooled sensitivity (95% CI: 54–87; *p* = 0.69) and a 70% pooled specificity (95% CI: 53–98; *p* = 0.01). The five studies published in 2017 and later had a 76% pooled sensitivity (95% CI: 63–89; *p* = 0.72) and a 68% pooled specificity (95% CI: 51–86; *p* = 0.00). See Table 3 for detailed results.

**Table 3:** Results of univariate meta-regression: sources of heterogeneity involved the PFI

Subgroup	Number of studies	Sensitivity [95% CI]	*p* value	Specificity [95% CI]	*p* value
Number of patients > 100					
Yes	5	70 (54–87)	0.69	70 (53–88)	< 0.01*
No	6	67 (50–84)	…	90 (81–98)	…
Published in 2017 and later					
Yes	5	76 (63–89)	0.72	68 (51–86)	< 0.01*
No	6	60 (44–77)	…	89 (81–97)	…
Number of readers > 1					
Yes	3	75 (53–97)	0.92	91 (80, 100)	0.51
No	8	67 (53–80)	…	77 (64–90)	…
Number of features > 1					
Yes	7	68 (52–83)	0.53	79 (65–94)	0.27
No	4	69 (50–89)	…	87 (73–100)	…

*95% CI* 95% confidence interval, *PFI* perinephric fat invasion

* *p* < 0.05, the difference was statistically significant

For imaging interpretation considering only the perinephric soft tissue density shadow-perinephric soft tissue stranding or nodules or both, the combined sensitivity, specificity, PLR, and NLR were 0.64 (95% CI: 0.40–0.83), 0.87 (95% CI: 0.70–0.95), 4.86 (95% CI: 2.27–10.44), and 0.41 (95% CI: 0.23–0.74), respectively (Supplementary Figs. 2a and 3). The area under the SROC curve was 0.83 (95% CI: 0.80–0.86) (Supplementary Fig. 2b). For imaging interpretation considering the perinephric soft tissue density shadow and other CT signs, including tumor margins, peritumoral vessels, increased density of perinephric fat et al, the combined sensitivity, specificity, PLR, and NLR were 0.72 (95% CI: 0.60–0.82), 0.74 (95% CI: 0.49–0.89), 2.74 (95% CI: 1.16–6.46), and 0.38 (95% CI: 0.21–0.67), respectively (Supplementary Figs. 2c and 4). The area under the SROC curve was 0.76 (95% CI: 0.72–0.80) (Supplementary Fig. 2d).

### CT assessment for RSFI

**Fig. 4:**
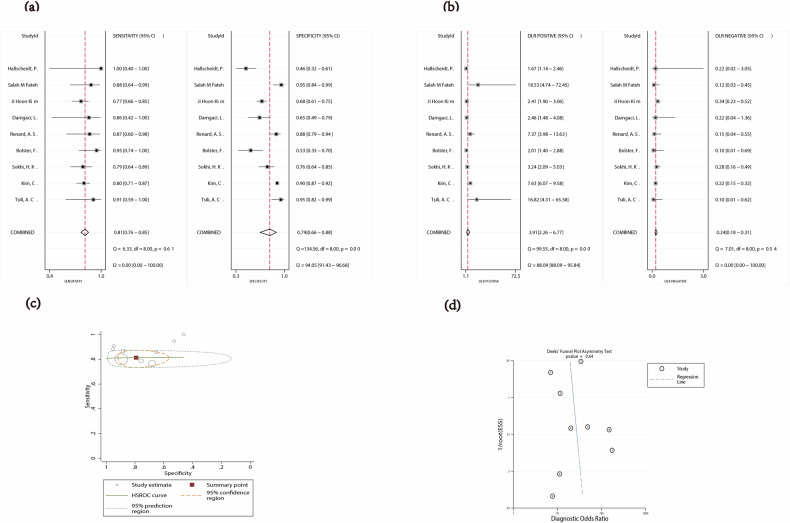
**a** Forest plot of sensitivity and specificity of CT assessment for RSFI. **b** Forest plot for likelihood ratio after combination (LR+, LR−). **c** The HSROC curve of CT assessment for RSFI. (HSROC hierarchical summary receiver operating characteristic). **d** The Deek funnel plot of studies on RSFI

The statistical analysis revealed significant heterogeneity in the specificity (*p* < 0.05, *I*^2^ = 94.05%) of CT assessments for RSFI, with pooled sensitivity at 0.81 (95% CI: 0.76–0.85) and pooled specificity at 0.79 (95% CI: 0.66–0.88), as depicted in Fig. 4a. The combined metrics of diagnostic performance, including the PLR of 3.91 (95% CI: 2.26–6.77), NLR of 0.24 (95% CI: 0.18–0.31), DOR of 16.57 (95% CI: 7.81–35.18), and diagnostic score of 2.81 (95% CI: 2.06–3.56), are illustrated in Fig. 4b and Supplementary Fig. 5. The area under the SROC curve was 0.82 (95% CI: 0.78–0.85), shown in Fig. 4c. The accuracy parameter (Lambda) was 13.30 (95% CI: −185.12 to 211.73).

Univariate meta-regression indicated that publication year, number of patients, number of signs, consensus read, and excretory phase significantly impacted the diagnostic performance. Among them, publication year, consensus read, and number of patients showed a significant effect on sensitivity, and the excretory phase showed a mild effect. The four studies published in 2017 and later had an 80% pooled sensitivity (95% CI: 72–87; *p* = 0.00) and an 82% pooled specificity (95% CI: 66–97; *p* = 0.87). The three studies published with consensus had an 80% pooled sensitivity (95% CI: 74–85; *p* = 0.00) and an 86% pooled specificity (95% CI: 73–99; *p* = 0.76). The three studies published with 100 or more cases had a 79% pooled sensitivity (95% CI: 73–84; *p* = 0.00) and an 80% pooled specificity (95% CI: 61–98; *p* = 0.65). The three studies published with the excretory phase had an 82% pooled sensitivity (95% CI: 75–90; *p* = 0.03) and a 90% pooled specificity (95% CI: 86–94; *p* = 0.72). Detailed results are available in Table 4.

**Table 4:** Results of univariate meta-regression: sources of heterogeneity involved the RSFI

Subgroup	Number of studies	Sensitivity [95% CI]	*p* value	Specificity [95% CI]	*p* value
Number of patients > 100					
Yes	3	79 (73–84)	< 0.001*	80 (61–98)	0.65
No	6	90 (84–97)	…	79 (65–93)	…
Published in 2017 and later					
Yes	4	80 (72–87)	< 0.001*	82 (67–97)	0.87
No	5	82 (76–89)	…	76 (60–93)	…
Number of Readers > 1					
Yes	7	81 (76–86)	0.11	77 (64–91)	0.45
No	2	88 (74–100)	…	85 (65–100)	…
Number of Features > 1					
Yes	5	82 (76–88)	< 0.001*	80 (65–94)	0.64
No	4	80 (73–88)	…	78 (61–96)	…
Consensus Read					
Yes	3	80 (74–85)	< 0.001	86 (73–99)	0.76
No	6	85 (78–92)	…	74 (59–89)	…
Excretory Phase					
Yes	4	82 (75–90)	< 0.05*	90 (86–94)	0.72
No	5	83 (74–92)	…	63 (55–71)	…

*95% CI* 95% confidence interval, *RSFI* renal sinus fat invasion

* *p* < 0.05, the difference was statistically significant

For imaging interpretation considering only the signs related to renal sinus structure, the combined sensitivity, specificity, PLR, and NLR were 0.91 (95% CI: 0.79–0.96), 0.83 (95% CI: 0.57–0.95), 5.38 (95% CI: 1.81–16.05), and 0.11 (95% CI: 0.05–0.26), respectively (Supplementary Figs. 6a and 7). The area under the SROC curve was 0.92 (95% CI: 0.90–0.94) (Supplementary Fig. 6b). For imaging interpretation considering the other signs (not related to renal sinus structure), the combined sensitivity, specificity, PLR, and NLR were 0.79 (95% CI: 0.71–0.85), 0.72 (95% CI: 0.55–0.84), 2.81 (95% CI: 1.60–4.93), and 0.29 (95% CI: 0.19–0.45), respectively (Supplementary Figs. 6c and 8). The area under the SROC curve was 0.81 (95% CI: 0.77–0.84) (Supplementary Fig. 6d).

### Publication bias

The Deek’s funnel plot of studies on PFI and RSFI showed a slope coefficient of 0.03 and 0.64, respectively, which indicate that there was mild publication bias in the included studies on PFI and no publication bias in the included studies on RSFI (Figs. 3d and 4d).

## Discussion

In this systematic review and meta-analysis assessing the diagnostic performance of CT for extrarenal fat invasion in RCC patients, CT and specific CT features demonstrated acceptable accuracy. However, the PLR was found to be low. We observed that perinephric soft tissue density shadows exhibited higher specificity for detecting PFI, while features related to the renal sinus structure indicated higher accuracy for RSFI. These findings underscore the potential of CT in evaluating PFI and RSFI in RCC patients. However, the limited number of positive samples in the current studies diminishes the reliability of these results, given that AUC is insensitive to imbalanced samples. Consequently, further large-scale trials are essential to substantiate these findings.

Pooled results provide valuable insights; however, the true sensitivity and specificity of CT for RCC could differ significantly from these findings due to wide confidence intervals and a limited number of studies. This highlights the challenges in identifying sources of heterogeneity and emphasizes the need for more comprehensive studies with larger samples of positive cases to verify the diagnostic capabilities of CT in RCC accurately. The studies included varied imaging criteria for evaluating PFI and RSFI. Due to the limited number of relevant studies, we can broadly classify the imaging signs for PFI into two categories: perinephric soft tissue density shadows (stranding or nodules) and combinations of these shadows with other CT signs (peritumoral vascularity, increased density of perinephric fat, and irregular tumor margins). The analysis revealed that the diagnostic efficacy of the first category was generally higher, with greater accuracy (0.83 vs 0.76) and specificity (0.87 vs 0.74), but lower sensitivity (0.64 vs 0.72). Various specific imaging signs are useful for preoperative assessment of PFI, including larger tumor size, perinephric soft tissue stranding, contrast-enhancing soft-tissue nodules, peritumoral vascularity, increased density of perinephric fat, and irregular tumor margins, all of which show varying diagnostic performances [11, 13, 27, 32, 34, 35, 40, 41]. Typically, perirenal soft tissue stranding exhibits high sensitivity but low specificity due to diverse underlying pathological mechanisms, from inflammation to neoplastic infiltration; the specificity of perinephric contrast-enhancing soft-tissue nodules and irregular tumor margins is high, notably so for the former, but their sensitivity is lower, limiting clinical application [27, 32, 33]. We combined perirenal soft tissue stranding with enhanced nodules into a subcategory, which affected the overall sensitivity adversely due to the low sensitivity of the nodules, similarly impacting specificity. For RSFI, due to scant research, we tentatively categorize the signs into two subtypes: those related to renal sinus structure (elongation of the tumor into the renal sinus, invasion, or compression of the pelvicalyceal system) and other signs (ill-defined or irregular tumor margins, enhancing tumor tissue within the sinus fat, small hyperdense strands surrounding a sinus lesion near the pelvicalyceal system, and finger-like projections). Prior studies suggest that signs related to renal sinus structure are more suitable as primary indicators, while other signs serve as supplemental [28, 39]. The diagnostic performance of the primary signs is superior, particularly for invasions of the pelvicalyceal system, and more stable for the supplemental signs. Our analysis confirms that the primary signs have significantly higher diagnostic efficacy, with better sensitivity (0.91 vs 0.79), specificity (0.83 vs 0.72), and accuracy (0.92 vs 0.81). Therefore, relying solely on single imaging signs is insufficient for accurate evaluation, and a judicious combination of signs requires further investigation.

Although the diagnostic performance of CT for extrarenal fat invasion remains inconclusive, this meta-analysis established a foundation for future research and clinical practice, such as furnishing a theoretical basis for the diagnostic accuracy of CT for extrarenal fat invasion and identifying useful imaging features with genuine diagnostic efficacy. The diagnostic capability of CT relies on identifiable features of PFI and RSFI. However, standardized CT characteristics for these conditions have not yet been established. Based on current research, further high-quality studies are necessary to identify specific imaging signs that could be integrated into the diagnostic system, and additional meta-analyses are needed to consolidate these findings. As part of the diagnostic system, exploring whether combinations of different signs can compensate for each other’s diagnostic limitations is also a valuable research direction. Modern CT machines equipped with more detectors and thinner collimators can produce high-resolution images [42]. Advanced technologies like photon-counting detector CT can enhance spatial resolution and improve iodine contrast signals, alone or in combination with virtual monoenergetic images, which are particularly useful in detecting small objects such as peritoneal implants [43, 44]. Future research should investigate whether CT scanners with more rows and higher spatial resolution can enhance the diagnostic accuracy for extrarenal fat invasion. The outcomes of such studies may also determine which imaging protocols and new techniques are most effective for assessment. Additionally, quantitative image analysis techniques, such as radiomics and deep learning, are promising areas for future exploration. Radiomics, in particular, has gained significant attention for its potential in personalized oncology management [11]. In RCC, applying machine learning to analyze first- and second-order texture structures extracted from renal masses has proven valuable [45–47]. However, the application of radiomics for predicting PFI and RSFI status in RCC patients is still rare. So far, only one study by Liu et al has demonstrated that a radiomics model, based on semi-automatic segmentation of RCC, could achieve higher predictive accuracy for PFI than traditional human readings (0.93 vs 0.80) [11].

This study has several limitations. Firstly, high heterogeneity complicates the interpretation of the pooled results. The included studies varied in design, including differences in imaging parameters such as the number of CT rows, enhancement phases, and CT slice thickness, as well as in diagnostic criteria, the proportion of positive cases, and the number of patients, all of which may contribute to observed heterogeneity. While we attempted to address this limitation through meta-regression analysis, the results must be interpreted with caution due to incomplete reporting of relevant features in the included studies. Secondly, a high or unclear risk of bias in the included studies limited the clinical applicability of the pooled results. Through rigorous exclusion and inclusion criteria, we selected studies that provided sufficient data to calculate the sensitivity and specificity of CT for assessing PFI. Thirdly, the small number of included studies, while sufficient to conduct a meta-analysis, restricted the feasibility of subgroup analyses and, in some cases, made them impossible. Moreover, the CT signs used across various studies differed, and the scarcity of relevant research prevented a meta-analysis based on consistent signs; instead, we can only broadly categorize and merge them. Fourthly, the low proportion of positive samples per study resulted in high pooled accuracy but low PLR, thereby diminishing the overall diagnostic performance of CT in evaluating extrarenal fat invasion. Fifthly, whether there was a buffer period was not specified in most of the included studies during the radiologists’ evaluation of PFI and RSFI. As a result, the evaluation outcomes may be influenced by each other. This warranted further exploration and improvement in future studies through more precise perspective multireader multicase (MRMC) research.

## Conclusion

In conclusion, the available evidence indicates that CT can effectively detect PFI and RSFI in patients with RCC, though this comes with considerable heterogeneity. However, the diagnostic performance of CT and its associated features is limited by slightly reduced accuracy, primarily due to the small number of positive cases in the existing studies. Additionally, the current PLR is low. Given these shortcomings, further large-scale trials involving non-AUC diagnostic evaluation parameters, such as positive predictive value, or matched positive and negative samples are essential to accurately determine the true diagnostic ability of CT in detecting extrarenal fat invasion.

## Supplementary information

ELECTRONIC SUPPLEMENTARY MATERIAL

## Data Availability

All data generated or analyzed during this study are included in this published article.

## Abbreviations

CT: Computed tomography
HSROC: Hierarchical summary receiver operating characteristic
NLR: Negative likelihood ratio
PFI: Perinephric fat invasion
PLR: Positive likelihood ratio
QUADAS-2: Quality assessment of diagnostic accuracy studies-2
RCC: Renal cell carcinoma
RSFI: Renal sinus fat invasion
SROC: Summary receiver operating characteristic
