# Supplementary material for: Diagnostic performance of CT for extrarenal fat invasion in renal cell carcinoma: a meta-analysis and systematic review
Source: Insights Imaging. 2025 Jan 15;16:19. doi: 10.1186/s13244-024-01889-0 (PMC11735820; doi:10.1186/s13244-024-01889-0)

Diagnostic performance of CT for extrarenal fat invasion in renal cell carcinoma: a meta-analysis and systematic review

ELECTRONIC SUPPLEMENTARY MATERIAL

Supplementary Table 1 Search Strategy

Updated	January 14th,2024
Databases used	PubMed, Web of Science, EMBASE and Cochrane Library databases
Medical subject heading (MeSH) terms and free terms used	
1.MesH terms: Carcinoma, Renal Cell<br>Free terms: Carcinomas, Renal Cell, Renal Cell Carcinomas, Nephroid Carcinoma, Carcinoma, Nephroid, Nephroid Carcinomas, Adenocarcinoma Of Kidney, Adenocarcinoma Of Kidneys, Kidney, Adenocarcinoma Of, Renal Cell Carcinoma, Renal Cell Cancer, Cancer, Renal Cell, Renal Cell Cancers, Adenocarcinoma, Renal, Renal Adenocarcinoma, Renal Adenocarcinomas, Renal Carcinoma, Carcinoma, Renal, Renal Carcinomas, Adenocarcinoma, Renal Cell, Adenocarcinomas, Renal Cell, Renal Cell Adenocarcinoma, Renal Cell Adenocarcinomas, Chromophobe Renal Cell Carcinoma, Sarcomatoid Renal Cell Carcinoma, Papillary Renal Cell Carcinoma, Renal Cell Carcinoma, Papillary, Chromophil Renal Cell Carcinoma, Clear Cell Renal Cell Carcinoma, Grawitz Tumor, Tumor, Grawitz, Clear Cell Renal Carcinoma, Carcinoma, Hypernephroid, Hypernephroid Carcinoma, Hypernephroid Carcinomas, Hypernephroma, Hypernephromas, Collecting Duct Carcinoma (Kidney), Carcinoma, Collecting Duct (Kidney), Carcinomas, Collecting Duct (Kidney), Collecting Duct Carcinomas (Kidney), Collecting Duct Carcinoma of the Kidney, Renal Collecting Duct Carcinoma, Collecting Duct Carcinoma, Carcinoma, Collecting Duct, Carcinomas, Collecting Duct, Collecting Duct Carcinomas	
2.MesH terms: No<br>Free terms: extrarenal fat	
3.MesH terms: No<br>Free terms: extrarenal fat invasion	
4.MesH terms: No<br>Free terms: perinephric fat	
5.MesH terms: No<br>Free terms: perirenal fat	
6.MesH terms: No<br>Free terms: perinephric fat invasion	
7.MesH terms: No	

Free terms: perirenal fat invasion
8.MesH terms: No<br>Free terms: renal sinus fat
9.MesH terms: No<br>Free terms: renal sinus fat invasion
10.MesH terms: Neoplasm Staging<br>Free terms: Staging, Neoplasm, Tumor Staging, Staging, Tumor, Cancer Staging, Staging, Cancer, TNM Staging, Staging, TNM, TNM Staging System, Staging System, TNM, Staging Systems, TNM, System, TNM Staging, Systems, TNM Staging, TNM Staging Systems, TNM Classification, Classification, TNM, Classifications, TNM, TNM Classifications
11.MesH terms: Tomography, X-Ray Computed<br>Free terms: X-Ray Computed Tomography, Tomography, X-Ray Computerized, Tomography, X Ray Computerized, Computed X Ray Tomography, X-Ray Computer Assisted Tomography, X Ray Computer Assisted Tomography, Tomography, X-Ray Computer Assisted, Tomography, X Ray Computer Assisted, Computerized Tomography, X Ray, Computerized Tomography, X-Ray, X-Ray Computerized Tomography, CT X Ray, CT X Rays, X Ray, CT, X Rays, CT, Tomodensitometry, Tomography, X Ray Computed, X Ray Tomography, Computed, X-Ray Tomography, Computed, Computed X-Ray Tomography, Tomographies, Computed X-Ray, Tomography, Computed X-Ray, Tomography, Xray Computed, Computed Tomography, Xray, Xray Computed Tomography, CAT Scan, X Ray, CAT Scan, X-Ray, CAT Scans, X-Ray, Scan, X-Ray CAT, Scans, X-Ray CAT, X-Ray CAT Scan, X-Ray CAT Scans, Tomography, Transmission Computed, Computed Tomography, Transmission, Transmission Computed Tomography, CT Scan, X-Ray, CT Scan, X Ray, CT Scans, X-Ray, Scan, X-Ray CT, Scans, X-Ray CT, X-Ray CT Scan, X-Ray CT Scans, Computed Tomography, X-Ray, Computed Tomography, X Ray, X Ray Computerized Tomography, Cine-CT, Cine CT, Electron Beam Computed Tomography, Electron Beam Tomography, Beam Tomography, Electron, Tomography, Electron Beam, Tomography, X-Ray Computerized Axial, Tomography, X Ray Computerized Axial, X-Ray Computerized Axial Tomography, X Ray Computerized Axial Tomography

The full search string for PubMed
(((("Tomography, X-Ray Computed"[Mesh]) OR (((X-Ray Computed Tomography[Title/Abstract]) OR (Tomography, X-Ray Computerized[Title/Abstract])) OR (Tomography, X Ray Computerized[Title/Abstract])) OR (Computed X Ray Tomography[Title/Abstract])) OR (X-Ray Computer Assisted Tomography[Title/Abstract])) OR (X Ray Computer Assisted Tomography[Title/Abstract])) OR (Tomography, X-Ray Computer Assisted[Title/Abstract])) OR (Tomography, X Ray Computer Assisted[Title/Abstract])) OR (Computerized Tomography, X Ray[Title/Abstract])) OR (Computerized Tomography, X-Ray[Title/Abstract])) OR (X-Ray Computerized Tomography[Title/Abstract])) OR (CT X Ray[Title/Abstract])) OR (CT X Rays[Title/Abstract])) OR (X Ray, CT[Title/Abstract])) OR (X Rays, CT[Title/Abstract])) OR (Tomodensitometry[Title/Abstract])) OR (Tomography, X Ray Computed[Title/Abstract])) OR (X Ray Tomography, Computed[Title/Abstract])) OR (X-Ray Tomography, Computed[Title/Abstract])) OR (Computed X-Ray Tomography[Title/Abstract])) OR (Tomographies, Computed X-Ray[Title/Abstract])) OR (Tomography, Computed X-Ray[Title/Abstract])) OR (Tomography, Xray Computed[Title/Abstract])) OR (Computed Tomography, Xray[Title/Abstract])) OR (Xray Computed Tomography[Title/Abstract])) OR (CAT Scan, X Ray[Title/Abstract])) OR (CAT Scan, X-Ray[Title/Abstract])) OR (CAT Scans, X-Ray[Title/Abstract])) OR (Scan, X-Ray CAT[Title/Abstract])) OR (Scans, X-Ray CAT[Title/Abstract])) OR (X-Ray CAT Scan[Title/Abstract])) OR (X-Ray CAT Scans[Title/Abstract])) OR (Tomography, Transmission Computed[Title/Abstract])) OR (Computed Tomography, Transmission[Title/Abstract])) OR (Transmission Computed Tomography[Title/Abstract])) OR (CT Scan, X-Ray[Title/Abstract])) OR (CT Scan, X Ray[Title/Abstract])) OR (CT Scans, X-Ray[Title/Abstract])) OR (Scan, X-Ray CT[Title/Abstract])) OR (Scans, X-Ray CT[Title/Abstract])) OR (X-Ray CT Scan[Title/Abstract])) OR (X-Ray CT Scans[Title/Abstract])) OR (Computed Tomography, X-Ray[Title/Abstract])) OR (Computed Tomography, X Ray[Title/Abstract])) OR (X Ray Computerized Tomography[Title/Abstract])) OR (Cine-CT[Title/Abstract])) OR (Cine CT[Title/Abstract])) OR (Electron Beam Computed Tomography[Title/Abstract])) OR (Electron Beam Tomography[Title/Abstract])) OR (Beam Tomography, Electron[Title/Abstract])) OR (Tomography, Electron Beam[Title/Abstract]))

OR (Tomography, X-Ray Computerized Axial[Title/Abstract])) OR (Tomography, X Ray Computerized Axial[Title/Abstract])) OR (X-Ray Computerized Axial Tomography[Title/Abstract])) OR (X Ray Computerized Axial Tomography[Title/Abstract]))) AND (("Carcinoma, Renal Cell"[Mesh]) OR ((Carcinomas, Renal Cell[Title/Abstract]) OR (Renal Cell Carcinomas[Title/Abstract])) OR (Nephroid Carcinoma[Title/Abstract])) OR (Carcinoma, Nephroid[Title/Abstract])) OR (Nephroid Carcinomas[Title/Abstract])) OR (Adenocarcinoma Of Kidney[Title/Abstract])) OR (Adenocarcinoma Of Kidneys[Title/Abstract])) OR (Kidney, Adenocarcinoma Of[Title/Abstract])) OR (Renal Cell Carcinoma[Title/Abstract])) OR (Renal Cell Cancer[Title/Abstract])) OR (Cancer, Renal Cell[Title/Abstract])) OR (Renal Cell Cancers[Title/Abstract])) OR (Adenocarcinoma, Renal[Title/Abstract])) OR (Renal Adenocarcinoma[Title/Abstract])) OR (Renal Adenocarcinomas[Title/Abstract])) OR (Renal Carcinoma[Title/Abstract])) OR (Carcinoma, Renal[Title/Abstract])) OR (Renal Carcinomas[Title/Abstract])) OR (Adenocarcinoma, Renal Cell[Title/Abstract])) OR (Adenocarcinomas, Renal Cell[Title/Abstract])) OR (Renal Cell Adenocarcinoma[Title/Abstract])) OR (Renal Cell Adenocarcinomas[Title/Abstract])) OR (Chromophobe Renal Cell Carcinoma[Title/Abstract])) OR (Sarcomatoid Renal Cell Carcinoma[Title/Abstract])) OR (Papillary Renal Cell Carcinoma[Title/Abstract])) OR (Renal Cell Carcinoma, Papillary[Title/Abstract])) OR (Chromophil Renal Cell Carcinoma[Title/Abstract])) OR (Clear Cell Renal Cell Carcinoma[Title/Abstract])) OR (Grawitz Tumor[Title/Abstract])) OR (Tumor, Grawitz[Title/Abstract])) OR (Clear Cell Renal Carcinoma[Title/Abstract])) OR (Carcinoma, Hypernephroid[Title/Abstract])) OR (Hypernephroid Carcinoma[Title/Abstract])) OR (Hypernephroid Carcinomas[Title/Abstract])) OR (Hypernephroma[Title/Abstract])) OR (Hypernephromas[Title/Abstract])) OR (Collecting Duct Carcinoma (Kidney[Title/Abstract]))) OR (Carcinoma, Collecting Duct (Kidney[Title/Abstract]))) OR (Carcinomas, Collecting Duct (Kidney[Title/Abstract]))) OR (Collecting Duct Carcinomas (Kidney[Title/Abstract]))) OR (Collecting Duct Carcinoma of the Kidney[Title/Abstract])) OR (Renal Collecting Duct Carcinoma[Title/Abstract])) OR (Collecting Duct Carcinoma[Title/Abstract])) OR (Carcinoma, Collecting Duct[Title/Abstract])) OR (Carcinomas, Collecting Duct[Title/Abstract])) OR (Collecting Duct Carcinomas[Title/Abstract])))) AND (((((((((extrarenal fat[Title/Abstract]) OR (extrarenal fat invasion[Title/Abstract])) OR (perirenal fat[Title/Abstract])) OR (perinephric fat[Title/Abstract])) OR (perinephric fat invasion[Title/Abstract])) OR (perirenal fat invasion[Title/Abstract])) OR (renal sinus fat[Title/Abstract])) OR (renal sinus fat invasion[Title/Abstract])) OR (("Neoplasm Staging"[Mesh]) OR (((((((((((((((((Staging, Neoplasm[Title/Abstract]) OR (Tumor Staging[Title/Abstract])) OR (Staging, Tumor[Title/Abstract])) OR (Cancer

Staging[Title/Abstract])) OR (Staging, Cancer[Title/Abstract])) OR (TNM Staging[Title/Abstract])) OR (Staging, TNM[Title/Abstract])) OR (TNM Staging System[Title/Abstract])) OR (Staging System, TNM[Title/Abstract])) OR (Staging Systems, TNM[Title/Abstract])) OR (System, TNM Staging[Title/Abstract])) OR (Systems, TNM Staging[Title/Abstract])) OR (TNM Staging Systems[Title/Abstract])) OR (TNM Classification[Title/Abstract])) OR (Classification, TNM[Title/Abstract])) OR (Classifications, TNM[Title/Abstract])) OR (TNM Classifications[Title/Abstract]))))) AND (sensitiv*[Title/Abstract] OR sensitivity and specificity[MeSH Terms] OR (predictive[Title/Abstract] AND value*[Title/Abstract]) OR predictive value of tests[MeSH Terms] OR accuracy*[Title/Abstract])

**The full search string for Embase**
'Carcinomas, Renal Cell':ab,ti OR 'Renal Cell Carcinomas':ab,ti OR 'Nephroid Carcinoma':ab,ti OR 'Carcinoma, Nephroid':ab,ti OR 'Nephroid Carcinomas':ab,ti OR 'Adenocarcinoma Of Kidney':ab,ti OR 'Adenocarcinoma Of Kidneys':ab,ti OR 'Kidney, Adenocarcinoma Of':ab,ti OR 'Renal Cell Carcinoma':ab,ti OR 'Renal Cell Cancer':ab,ti OR 'Cancer, Renal Cell':ab,ti OR 'Renal Cell Cancers':ab,ti OR 'Adenocarcinoma, Renal':ab,ti OR 'Renal Adenocarcinoma':ab,ti OR 'Renal Adenocarcinomas':ab,ti OR 'Renal Carcinoma':ab,ti OR 'Carcinoma, Renal':ab,ti OR 'Renal Carcinomas':ab,ti OR 'Adenocarcinoma, Renal Cell':ab,ti OR 'Adenocarcinomas, Renal Cell':ab,ti OR 'Renal Cell Adenocarcinoma':ab,ti OR 'Renal Cell Adenocarcinomas':ab,ti OR 'Chromophobe Renal Cell Carcinoma':ab,ti OR 'Sarcomatoid Renal Cell Carcinoma':ab,ti OR 'Papillary Renal Cell Carcinoma':ab,ti OR 'Renal Cell Carcinoma, Papillary':ab,ti OR 'Chromophil Renal Cell Carcinoma':ab,ti OR 'Clear Cell Renal Cell Carcinoma':ab,ti OR 'Grawitz Tumor':ab,ti OR 'Tumor, Grawitz':ab,ti OR 'Clear Cell Renal Carcinoma':ab,ti OR 'Carcinoma, Hypernephroid':ab,ti OR 'Hypernephroid Carcinoma':ab,ti OR 'Hypernephroid Carcinomas':ab,ti OR 'Hypernephroma':ab,ti OR 'Hypernephromas':ab,ti OR 'Collecting Duct Carcinoma (Kidney)':ab,ti OR 'Carcinoma, Collecting Duct (Kidney)':ab,ti OR 'Carcinomas, Collecting Duct (Kidney)':ab,ti OR 'Collecting Duct Carcinomas (Kidney)':ab,ti OR 'Collecting Duct Carcinoma of the Kidney':ab,ti OR 'Renal Collecting Duct Carcinoma':ab,ti OR 'Collecting Duct Carcinoma':ab,ti OR 'Carcinoma, Collecting Duct':ab,ti OR 'Carcinomas, Collecting Duct':ab,ti OR 'Collecting Duct Carcinomas':ab,ti AND 'extrarenal fat':ab,ti OR 'extrarenal fat invasion':ab,ti OR 'perinephric fat invasion':ab,ti OR 'perirenal fat invasion':ab,ti OR 'perinephric fat':ab,ti OR 'perirenal fat':ab,ti OR 'renal sinus fat invasion':ab,ti OR 'renal sinus fat':ab,ti OR 'Staging, Neoplasm':ab,ti OR 'Tumor Staging':ab,ti OR 'Staging, Tumor':ab,ti OR 'Cancer Staging':ab,ti OR 'Staging, Cancer':ab,ti OR 'TNM Staging':ab,ti OR 'Staging, TNM':ab,ti OR 'TNM Staging System':ab,ti OR 'Staging System, TNM':ab,ti OR 'Staging

Systems, TNM':ab,ti OR 'System, TNM Staging':ab,ti OR 'Systems, TNM Staging':ab,ti OR 'TNM Staging Systems':ab,ti OR 'TNM Classification':ab,ti OR 'Classification, TNM':ab,ti OR 'Classifications, TNM':ab,ti OR 'TNM Classifications':ab,ti AND 'X-Ray Computed Tomography':ab,ti OR 'Tomography, X-Ray Computerized':ab,ti OR 'Tomography, X Ray Computerized':ab,ti OR 'Computed X Ray Tomography':ab,ti OR 'X-Ray Computer Assisted Tomography':ab,ti OR 'X Ray Computer Assisted Tomography':ab,ti OR 'Tomography, X-Ray Computer Assisted':ab,ti OR 'Tomography, X Ray Computer Assisted':ab,ti OR 'Computerized Tomography, X Ray':ab,ti OR 'Computerized Tomography, X-Ray':ab,ti OR 'X-Ray Computerized Tomography':ab,ti OR 'CT X Ray':ab,ti OR 'CT X Rays':ab,ti OR 'X Ray, CT':ab,ti OR 'X Rays, CT':ab,ti OR 'Tomodensitometry':ab,ti OR 'Tomography, X Ray Computed':ab,ti OR 'X Ray Tomography, Computed':ab,ti OR 'X-Ray Tomography, Computed':ab,ti OR 'Computed X-Ray Tomography':ab,ti OR 'Tomographies, Computed X-Ray':ab,ti OR 'Tomography, Computed X-Ray':ab,ti OR 'Tomography, Xray Computed':ab,ti OR 'Computed Tomography, Xray':ab,ti OR 'Xray Computed Tomography':ab,ti OR 'CAT Scan, X Ray':ab,ti OR 'CAT Scan, X-Ray':ab,ti OR 'CAT Scans, X-Ray':ab,ti OR 'Scan, X-Ray CAT':ab,ti OR 'Scans, X-Ray CAT':ab,ti OR 'X-Ray CAT Scan':ab,ti OR 'X-Ray CAT Scans':ab,ti OR 'Tomography, Transmission Computed':ab,ti OR 'Computed Tomography, Transmission':ab,ti OR 'Transmission Computed Tomography':ab,ti OR 'CT Scan, X-Ray':ab,ti OR 'CT Scan, X Ray':ab,ti OR 'CT Scans, X-Ray':ab,ti OR 'Scan, X-Ray CT':ab,ti OR 'Scans, X-Ray CT':ab,ti OR 'X-Ray CT Scan':ab,ti OR 'X-Ray CT Scans':ab,ti OR 'Computed Tomography, X-Ray':ab,ti OR 'Computed Tomography, X Ray':ab,ti OR 'X Ray Computerized Tomography':ab,ti OR 'Cine-CT':ab,ti OR 'Cine CT':ab,ti OR 'Electron Beam Computed Tomography':ab,ti OR 'Electron Beam Tomography':ab,ti OR 'Beam Tomography, Electron':ab,ti OR 'Tomography, Electron Beam':ab,ti OR 'Tomography, X-Ray Computerized Axial':ab,ti OR 'Tomography, X Ray Computerized Axial':ab,ti OR 'X-Ray Computerized Axial Tomography':ab,ti OR 'X Ray Computerized Axial Tomography':ab,ti AND 'sensitiv':ab,ti OR 'sensitivity and specificity':ab,ti OR 'predictive':ab,ti OR 'predictive value of tests':ab,ti OR 'accuracy':ab,ti

The full search string for Cochrane Library databases
(Carcinomas, Renal Cell):ab,ti,kw OR (Renal Cell Carcinomas):ab,ti,kw OR (Nephroid Carcinoma):ab,ti,kw OR (Carcinoma, Nephroid):ab,ti,kw OR (Nephroid Carcinomas):ab,ti,kw OR (Adenocarcinoma Of Kidney):ab,ti,kw OR (Adenocarcinoma Of Kidneys):ab,ti,kw OR (Kidney, Adenocarcinoma Of):ab,ti,kw OR (Renal Cell Carcinoma):ab,ti,kw OR (Renal Cell Cancer):ab,ti,kw OR (Cancer, Renal Cell):ab,ti,kw OR (Renal Cell Cancers):ab,ti,kw OR (Adenocarcinoma, Renal):ab,ti,kw OR (Renal Adenocarcinoma):ab,ti,kw OR (Renal Adenocarcinomas):ab,ti,kw OR (Renal Carcinoma):ab,ti,kw OR (Carcinoma, Renal):ab,ti,kw OR (Renal Carcinomas):ab,ti,kw OR (Adenocarcinoma, Renal Cell):ab,ti,kw OR (Adenocarcinomas, Renal Cell):ab,ti,kw OR (Renal Cell Adenocarcinoma):ab,ti,kw OR (Renal Cell Adenocarcinomas):ab,ti,kw OR (Chromophobe Renal Cell Carcinoma):ab,ti,kw OR (Sarcomatoid Renal Cell Carcinoma):ab,ti,kw OR (Papillary Renal Cell Carcinoma):ab,ti,kw OR (Renal Cell Carcinoma, Papillary):ab,ti,kw OR (Chromophil Renal Cell Carcinoma):ab,ti,kw OR (Clear Cell Renal Cell Carcinoma):ab,ti,kw OR (Grawitz Tumor):ab,ti,kw OR (Tumor, Grawitz):ab,ti,kw OR (Clear Cell Renal Carcinoma):ab,ti,kw OR (Carcinoma, Hypernephroid):ab,ti,kw OR (Hypernephroid Carcinoma):ab,ti,kw OR (Hypernephroid Carcinomas):ab,ti,kw OR (Hypernephroma):ab,ti,kw OR (Hypernephromas):ab,ti,kw OR (Collecting Duct Carcinoma (Kidney)):ab,ti,kw OR (Carcinoma, Collecting Duct (Kidney)):ab,ti,kw OR (Carcinomas, Collecting Duct (Kidney)):ab,ti,kw OR (Collecting Duct Carcinomas (Kidney)):ab,ti,kw OR (Collecting Duct Carcinoma of the Kidney):ab,ti,kw OR (Renal Collecting Duct Carcinoma):ab,ti,kw OR (Collecting Duct Carcinoma):ab,ti,kw OR (Carcinoma, Collecting Duct):ab,ti,kw OR (Carcinomas, Collecting Duct):ab,ti,kw OR (Collecting Duct Carcinomas):ab,ti,kw AND (Staging, Neoplasm):ab,ti,kw OR (Tumor Staging):ab,ti,kw OR (Staging, Tumor):ab,ti,kw OR (Cancer Staging):ab,ti,kw OR (Staging, Cancer):ab,ti,kw OR (TNM Staging):ab,ti,kw OR (Staging, TNM):ab,ti,kw OR (TNM Staging System):ab,ti,kw OR (Staging System, TNM):ab,ti,kw OR (Staging Systems, TNM):ab,ti,kw OR (System, TNM Staging):ab,ti,kw OR (Systems, TNM Staging):ab,ti,kw OR (TNM Staging Systems):ab,ti,kw OR (TNM Classification):ab,ti,kw OR (Classification, TNM):ab,ti,kw OR (Classifications, TNM):ab,ti,kw OR (TNM Classifications):ab,ti,kw OR (extrarenal fat invasion):ab,ti,kw OR (extrarenal fat):ab,ti,kw OR (perinephric fat invasion):ab,ti,kw OR (perirenal fat invasion):ab,ti,kw OR (perinephric fat):ab,ti,kw OR (perirenal fat):ab,ti,kw OR (renal sinus fat invasion):ab,ti,kw OR (renal sinus fat):ab,ti,kw AND (X-Ray Computed Tomography):ab,ti,kw OR (Tomography, X-Ray Computerized):ab,ti,kw OR (Tomography, X Ray Computerized):ab,ti,kw OR (Computed X Ray Tomography):ab,ti,kw OR (X-Ray Computer Assisted Tomography):ab,ti,kw OR (X Ray Computer Assisted Tomography):ab,ti,kw

OR (Tomography, X-Ray Computer Assisted):ab,ti,kw OR (Tomography, X Ray Computer Assisted):ab,ti,kw OR (Computerized Tomography, X Ray):ab,ti,kw OR (Computerized Tomography, X-Ray):ab,ti,kw OR (X-Ray Computerized Tomography):ab,ti,kw OR (CT X Ray):ab,ti,kw OR (CT X Rays):ab,ti,kw OR (X Ray, CT):ab,ti,kw OR (X Rays, CT):ab,ti,kw OR (Tomodensitometry):ab,ti,kw OR (Tomography, X Ray Computed):ab,ti,kw OR (X Ray Tomography, Computed):ab,ti,kw OR (X-Ray Tomography, Computed):ab,ti,kw OR (Computed X-Ray Tomography):ab,ti,kw OR (Tomographies, Computed X-Ray):ab,ti,kw OR (Tomography, Computed X-Ray):ab,ti,kw OR (Tomography, Xray Computed):ab,ti,kw OR (Computed Tomography, Xray):ab,ti,kw OR (Xray Computed Tomography):ab,ti,kw OR (CAT Scan, X Ray):ab,ti,kw OR (CAT Scan, X-Ray):ab,ti,kw OR (CAT Scans, X-Ray):ab,ti,kw OR (Scan, X-Ray CAT):ab,ti,kw OR (Scans, X-Ray CAT):ab,ti,kw OR (X-Ray CAT Scan):ab,ti,kw OR (X-Ray CAT Scans):ab,ti,kw OR (Tomography, Transmission Computed):ab,ti,kw OR (Computed Tomography, Transmission):ab,ti,kw OR (Transmission Computed Tomography):ab,ti,kw OR (CT Scan, X-Ray):ab,ti,kw OR (CT Scan, X Ray):ab,ti,kw OR (CT Scans, X-Ray):ab,ti,kw OR (Scan, X-Ray CT):ab,ti,kw OR (Scans, X-Ray CT):ab,ti,kw OR (X-Ray CT Scan):ab,ti,kw OR (X-Ray CT Scans):ab,ti,kw OR (Computed Tomography, X-Ray):ab,ti,kw OR (Computed Tomography, X Ray):ab,ti,kw OR (X Ray Computerized Tomography):ab,ti,kw OR (Cine-CT):ab,ti,kw OR (Cine CT):ab,ti,kw OR (Electron Beam Computed Tomography):ab,ti,kw OR (Electron Beam Tomography):ab,ti,kw OR (Beam Tomography, Electron):ab,ti,kw OR (Tomography, Electron Beam):ab,ti,kw OR (Tomography, X-Ray Computerized Axial):ab,ti,kw OR (Tomography, X Ray Computerized Axial):ab,ti,kw OR (X-Ray Computerized Axial Tomography):ab,ti,kw OR (X Ray Computerized Axial Tomography):ab,ti,kw AND (sensitiv):ab,ti,kw OR (sensitivity and specificity):ab,ti,kw OR (predictive):ab,ti,kw OR (predictive value of tests):ab,ti,kw OR (accuracy):ab,ti,kw OR

The full search string for Web of Science
Carcinoma, Renal Cell or Carcinomas, Renal Cell or Renal Cell Carcinomas or Nephroid Carcinoma or Carcinoma, Nephroid or Nephroid Carcinomas or Adenocarcinoma Of Kidney or Adenocarcinoma Of Kidneys or Kidney, Adenocarcinoma Of or Renal Cell Carcinoma or Renal Cell Cancer or Cancer, Renal Cell or Renal Cell Cancers or Adenocarcinoma, Renal or Renal Adenocarcinoma or Renal Adenocarcinomas or Renal Carcinoma or Carcinoma, Renal or Renal Carcinomas or Adenocarcinoma, Renal Cell or Adenocarcinomas, Renal Cell or Renal Cell Adenocarcinoma or Renal Cell Adenocarcinomas or Chromophobe Renal Cell Carcinoma or Sarcomatoid Renal Cell Carcinoma or Papillary Renal Cell Carcinoma or Renal Cell Carcinoma, Papillary or Chromophil Renal Cell Carcinoma or Clear Cell Renal Cell Carcinoma or Grawitz Tumor or Tumor, Grawitz or Clear Cell Renal Carcinoma or Carcinoma, Hypernephroid or Hypernephroid Carcinoma or Hypernephroid Carcinomas or Hypernephroma or Hypernephromas or Collecting Duct Carcinoma (Kidney) or Carcinoma, Collecting Duct (Kidney) or Carcinomas, Collecting Duct (Kidney) or Collecting Duct Carcinomas (Kidney) or Collecting Duct Carcinoma of the Kidney or Renal Collecting Duct Carcinoma or Collecting Duct Carcinoma or Carcinoma, Collecting Duct or Carcinomas, Collecting Duct or Collecting Duct Carcinomas AND Neoplasm Staging or Staging, Neoplasm or Tumor Staging or Staging, Tumor or Cancer Staging or Staging, Cancer or TNM Staging or Staging, TNM or TNM Staging System or Staging System, TNM or Staging Systems, TNM or System, TNM Staging or Systems, TNM Staging or TNM Staging Systems or TNM Classification or Classification, TNM or Classifications, TNM or TNM Classifications or extrarenal fat invasion or extrarenal fat or perinephric fat invasion or perirenal fat invasion or perinephric fat or perirenal fat or renal sinus fat invasion or renal sinus fat AND Tomography, X-Ray Computed or X-Ray Computed Tomography or Tomography, X-Ray Computerized or Tomography, X Ray Computerized or Computed X Ray Tomography or X-Ray Computer Assisted Tomography or X Ray Computer Assisted Tomography or Tomography, X-Ray Computer Assisted or Tomography, X Ray Computer Assisted or Computerized Tomography, X Ray or Computerized Tomography, X-Ray or X-Ray Computerized Tomography or CT X Ray or CT X Rays or X Ray, CT or X Rays, CT or Tomodensitometry or Tomography, X Ray Computed or X Ray Tomography, Computed or X-Ray Tomography, Computed or Computed X-Ray Tomography or Tomographies, Computed X-Ray or Tomography, Computed X-Ray or Tomography, Xray Computed or Computed Tomography, Xray or Xray Computed Tomography or CAT Scan, X Ray or CAT Scan, X-Ray or CAT Scans, X-Ray or Scan, X-Ray CAT or Scans, X-Ray CAT or X-Ray CAT Scan or X-Ray CAT Scans or Tomography, Transmission Computed or Computed Tomography, Transmission or Transmission Computed

Tomography or CT Scan, X-Ray or CT Scan, X Ray or CT Scans, X-Ray or Scan, X-Ray CT or Scans, X-Ray CT or X-Ray CT Scan or X-Ray CT Scans or Computed Tomography, X-Ray or Computed Tomography, X Ray or X Ray Computerized Tomography or Cine-CT or Cine CT or Electron Beam Computed Tomography or Electron Beam Tomography or Beam Tomography, Electron or Tomography, Electron Beam or Tomography, X-Ray Computerized Axial or Tomography, X Ray Computerized Axial or X-Ray Computerized Axial Tomography or X Ray Computerized Axial Tomography AND sensitiv or sensitivity and specificity or predictive or predictive value of tests or accuracy

Supplementary Table 3 The examination protocols of the included studies involved the PFI

First Author [Reference No.]	Year	number and timing of post-contrast measurements (s)	slice thickness of reformation （mm）	volume and flow rate of contrast material (mL, mL/s)
Liu et al. (24)	2012	3, 30s,60s,300s	-	120-200mL, 3mL/s
Tsili et al. (25)	2013	3, 25s,70s,240s	-	150mL, 3mL/s
Damgaci et al. (26)	2021	1, 100s	0.5mm	100mL, 3.5mL/s
Landman et al. (30)	2017	132 patients-3, 35s,70s,600s	3mm	80-100mL, -
		29 patients-1, -	3mm	120-140mL, -
Liu et al. (31)	2023	1, -	1mm	90mL, 3mL/s
Türkvatan et al. (32)	2009	2, 30s,120s	1.25mm,2.5mm	120mL, 3mL/s
Kim et al. (33)	2014	3, 60s,150-180s,270-400s	3mm,5mm	100-120mL, 3mL/s
Fateh et al. (34)	2023	2, -	1-2mm	-, 2-4mL/s
Johnson et al. (35)	1987	-, -	-	200mL, -
Sokhi et al. (40)	2015	1, -	-	100mL, 3mL/s
Renard et al. (41)	2019	3, -	-	-

Supplementary Table 4 The examination protocols of the included studies involved the RSFI

First Author [Reference No.]	Year	number and timing of post-contrast measurements (s)	slice thickness of reformation （mm）	volume and flow rate of contrast material (mL, mL/s)
Tsili et al. (25)	2013	3, 25s,70s,240s	-	150mL, 3mL/s
Damgaci et al. (26)	2021	1, 100s	0.5mm	100mL, 3.5mL/s
Fateh et al. (34)	2023	2, -	1-2mm	-, 2-4mL/s
Bolster et al. (36)	2016	2, -	-	90mL, 3.5mL/s
Hallscheidt et al. (37)	2006	2, 30s,120s	2mm,5mm	120mL,3 mL/s
Kim et al. (38)	2014	3, 60s,150-180s,270-400s	3mm,5mm	100-120mL, 3mL/s
Kim et al. (39)	2021	2, 30s,90s	3mm	100mL, 3-4mL/s
Sokhi et al. (40)	2015	1, -	-	100mL, 3mL/s
Renard et al. (41)	2019	3, -	-	-

Supplementary Figure 1 Forest plot for diagnostic odds ratio and diagnostic score for PFI after combination.

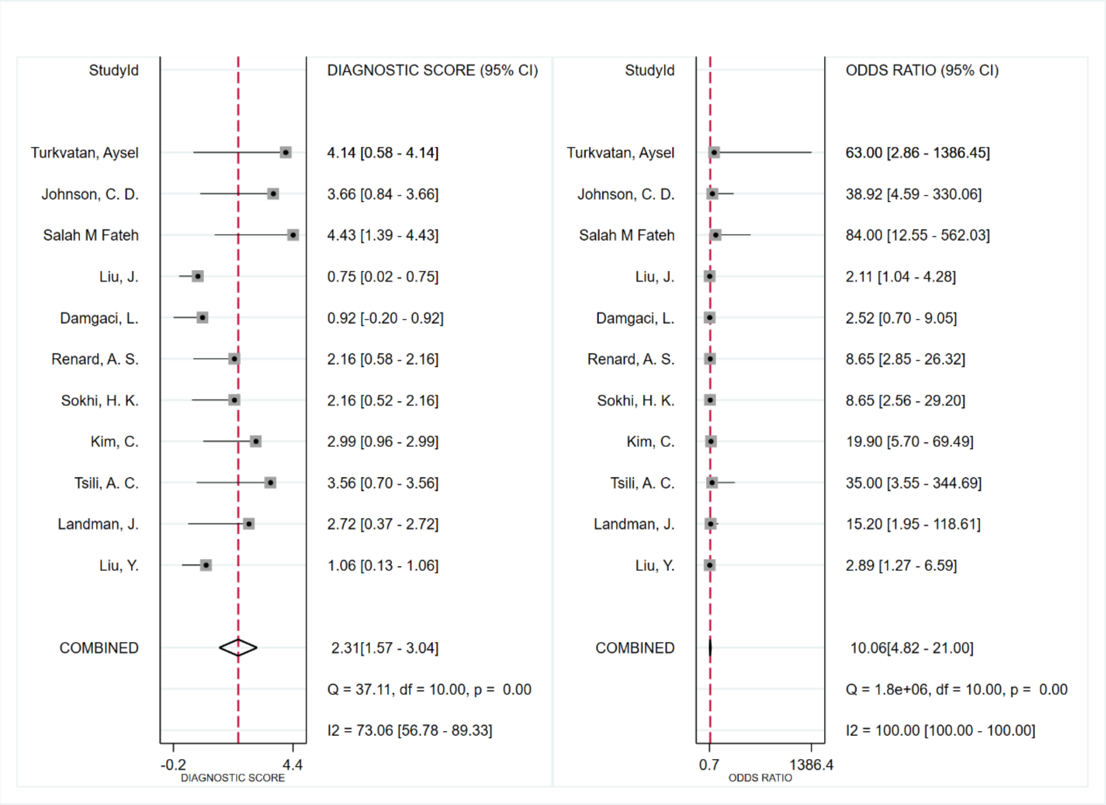

Supplementary Figure 2a Forest plot of sensitivity and specificity of the perinephric soft tissue density shadow assessment for PFI. **Figure 2b** The HSROC curve of the perinephric soft tissue density shadow assessment for PFI. **Figure 2c** Forest plot of sensitivity and specificity of the perinephric soft tissue density shadow and other signs assessment for PFI. **Figure 2d** The HSROC curve of the perinephric soft tissue density shadow and other signs assessment for PFI. (HSROC: Hierarchical summary receiver operating characteristic).

(a)

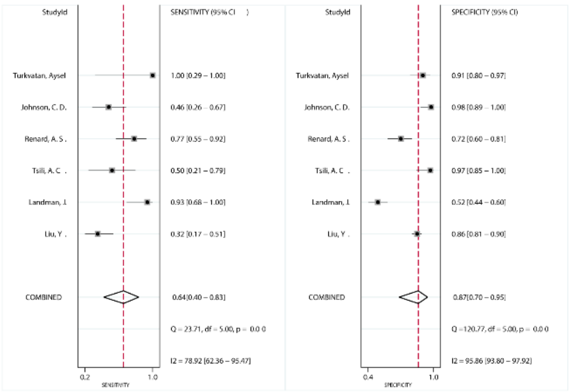

(b)

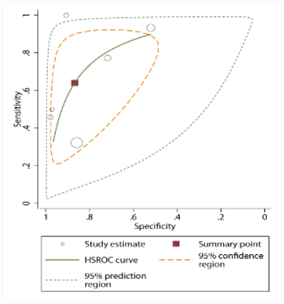

(c)

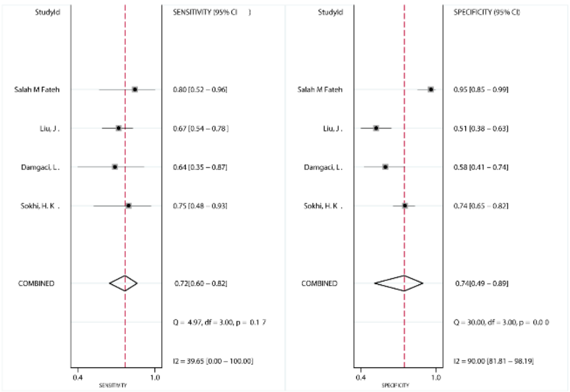

(d)

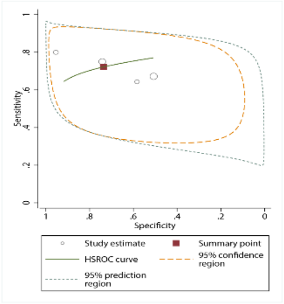

Supplementary Figure 3 Forest plot for likelihood ratio of the perinephric soft tissue density shadow assessment for PFI after combination (LR+, LR-).

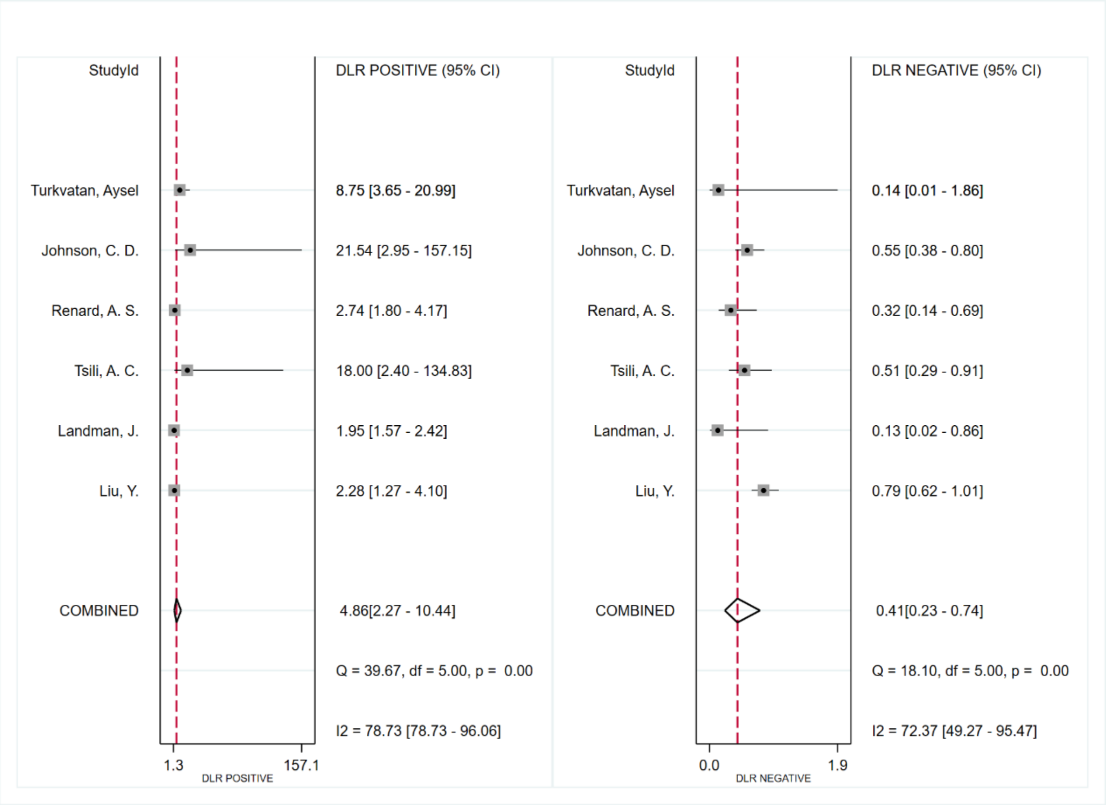

Supplementary Figure 4 Forest plot for likelihood ratio of the perinephric soft tissue density shadow and other signs assessment for PFI after combination (LR+, LR-).

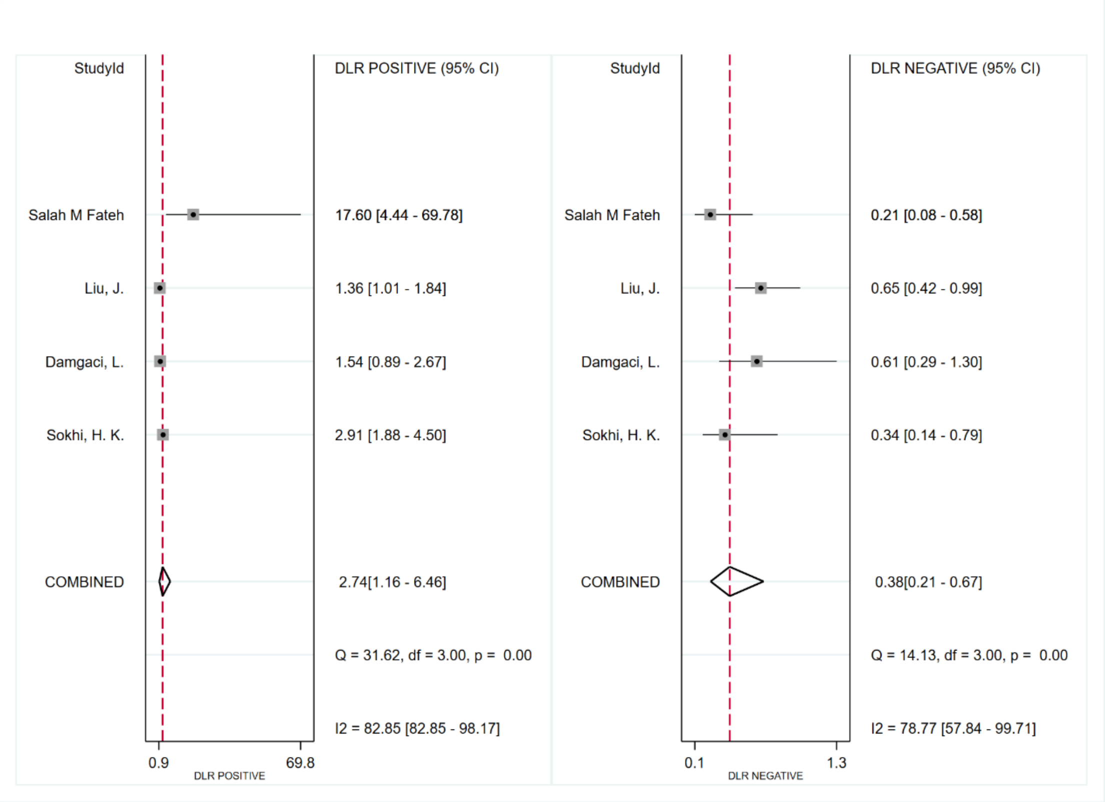

Supplementary Figure 5 Forest plot for diagnostic odds ratio and diagnostic score for RSFI after combination.

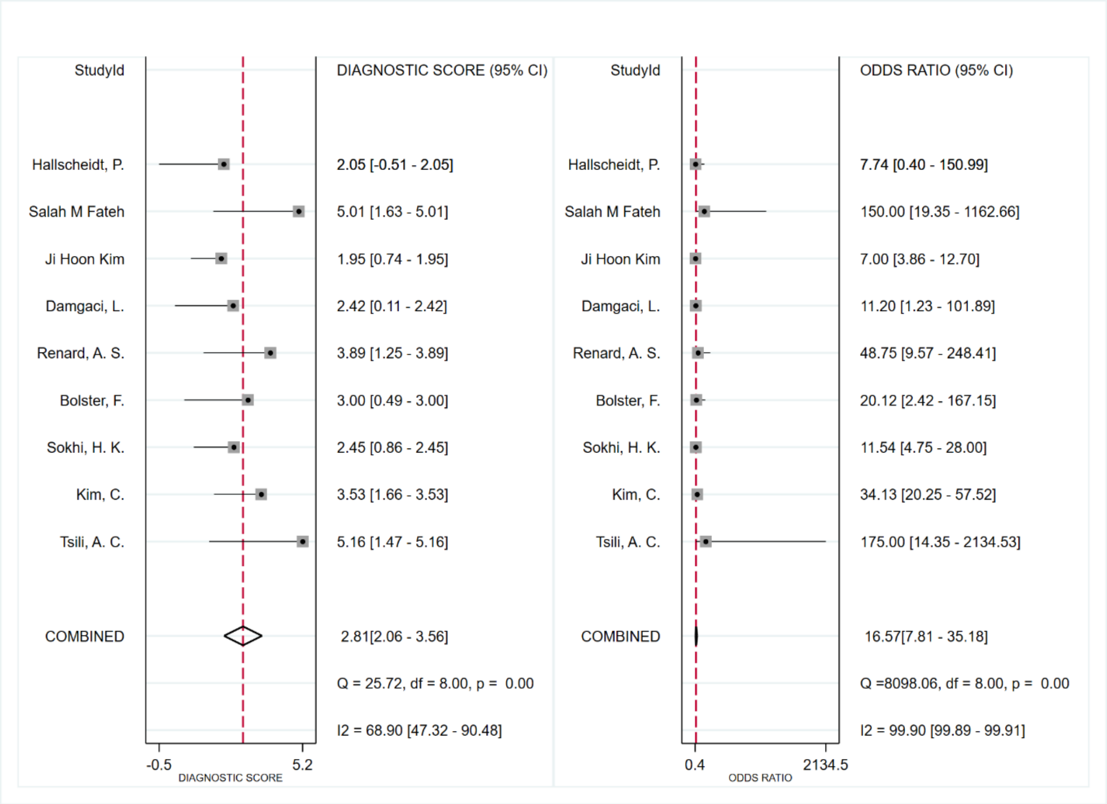

Supplementary Figure 6a Forest plot of sensitivity and specificity of the signs related to renal sinus structure assessment for RSFI. **Figure 6b** The HSROC curve of the signs related to renal sinus structure assessment for RSFI. **Figure 6c** Forest plot of sensitivity and specificity of the other signs assessment for RSFI. **Figure 6d** The HSROC curve of the other signs assessment for RSFI. (HSROC: Hierarchical summary receiver operating characteristic).

(a)

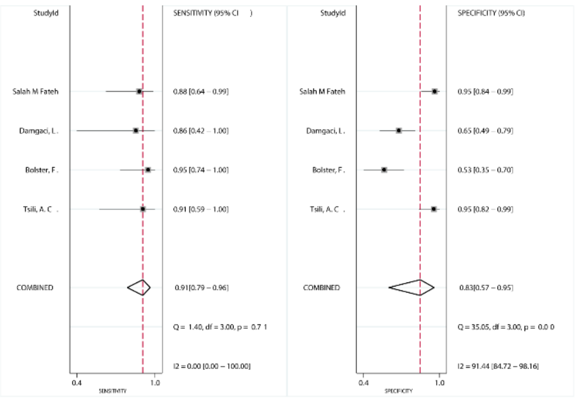

(b)

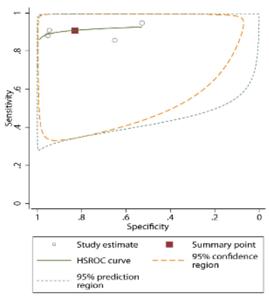

(c)

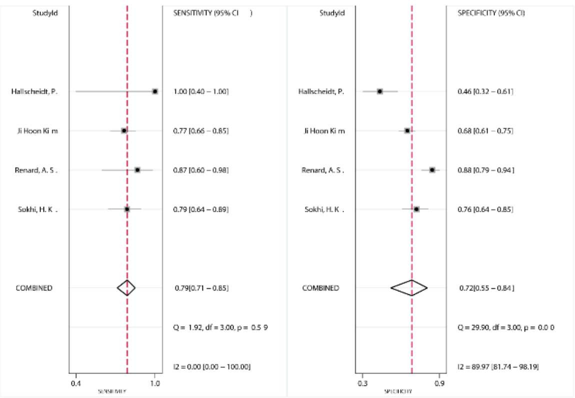

(d)

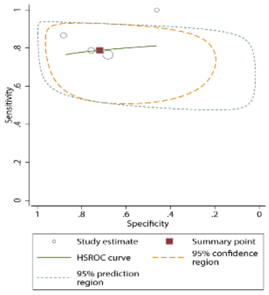

Supplementary Figure 7 Forest plot for likelihood ratio of the signs related to renal sinus structure assessment for PFI after combination (LR+, LR-).

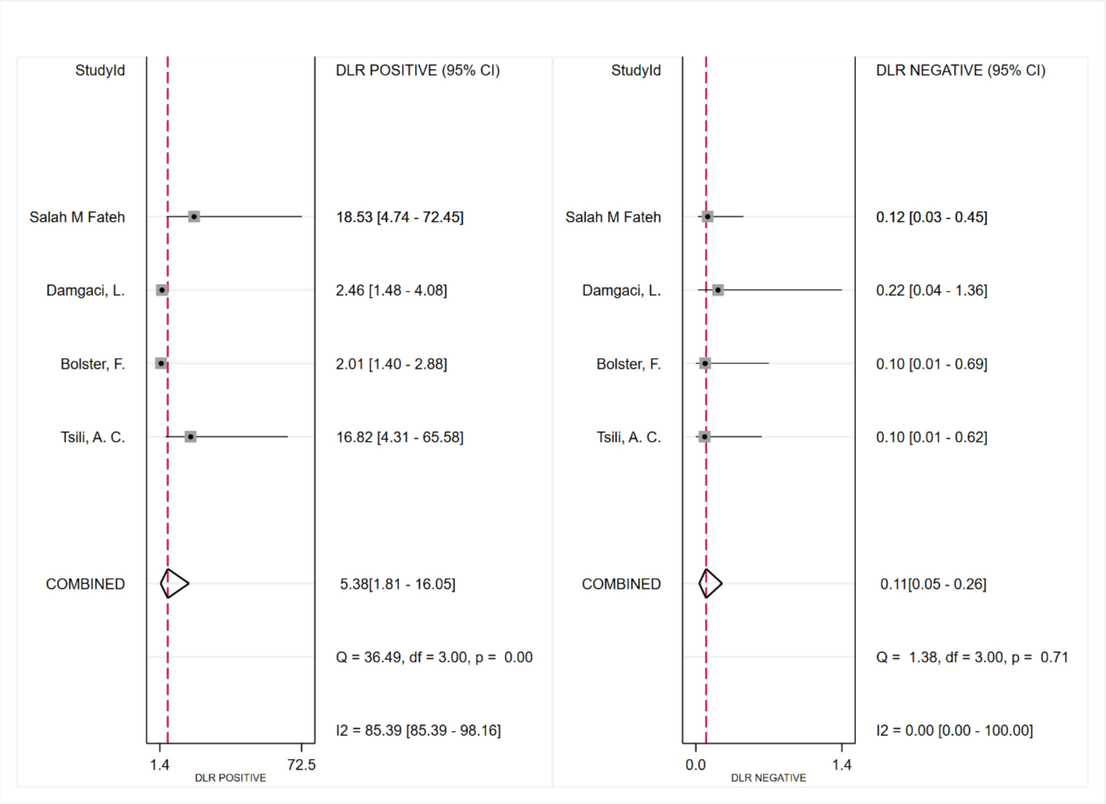

Supplementary Figure 8 Forest plot for likelihood ratio of the other signs assessment for PFI after combination (LR+, LR-).

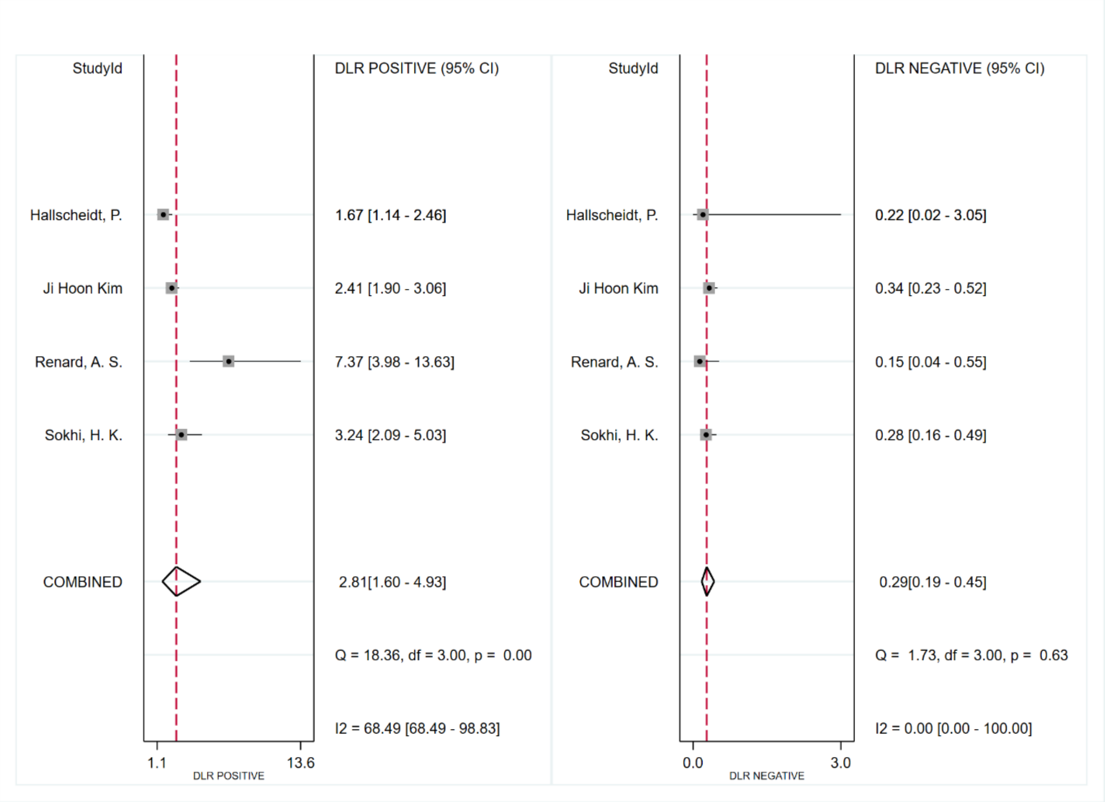

Supplement: Supplementary file 1 — ELECTRONIC SUPPLEMENTARY MATERIAL [file 13244_2024_1889_MOESM1_ESM.pdf]
